# Financial incentives and long-acting injectable antipsychotics engagement: community mental health professionals’ perspectives

**DOI:** 10.1186/s12888-025-07165-9

**Published:** 2025-08-18

**Authors:** Nathan Hodson, Madiha Majid, Ivo Vlaev, Swaran Singh

**Affiliations:** 1 Unit of Mental Health and Wellbeing Warwick Medical School, University of Warwick, Gibbet Hill Road, Coventry, CV4 7AL UK; 2 University of Southern California, Los Angeles, USA; 3 University of Nottingham, Nottingham, UK; 4 Centre for Behavioral and Implementation Science Interventions (BISI), Yong Loo Lin School of Medicine, National University of Singapore, Singapore, Republic of Singapore

**Keywords:** Financial incentives, Clinician perspectives, Attitudes, Adherence, Engagement, Acceptability

## Abstract

**Background:**

Long-Acting injectable (LAI) antipsychotics prevent relapse in patients with unreliable adherence to oral antipsychotics. Non-engagement with treatment is associated with relapse and involuntary admission. Financial incentives have been shown to increase engagement with LAI antipsychotics among those at highest risk of relapse. This study aimed to explore whether community mental health professionals who deliver LAI antipsychotics find financial incentives acceptable and whether empirical research has addressed their concerns.

**Methods:**

Fourteen mental health professionals who deliver LAI antipsychotics were interviewed after snowball recruitment. Each then saw a presentation outlining recent research findings into financial incentives for LAI antipsychotic engagement before interviews resumed. Interviews explored the advantages and challenges professionals anticipated with respect to financial incentives for LAIs. Thematic analysis was conducted and themes were compared with findings from the literature.

**Results:**

Median self-reported support increased from “5/10” to “6.75/10” after the information presentation. Participants responded positively to the idea that incentives could be seen as ‘rewards’. They readily drew connections between improvements in engagement, increased insight, and stronger therapeutic relationships. Practical implementation challenges were envisioned along with proposed solutions. Several participants remained concerned that financial incentives would lead to increased drug use.

**Conclusions:**

Mental health professionals who deliver LAI antipsychotics hold a range of views about the potential role of financial incentives to increase engagement. When professionals are aware of research findings they are more positive about incentives. Further research should consider patients’ and professionals’ preferences for the design of incentive regimes.

**Supplementary Information:**

The online version contains supplementary material available at 10.1186/s12888-025-07165-9.

## Introduction

Long-acting injectable (LAI) antipsychotic therapy is recommended for patients with psychosis or schizophrenia who “prefer such treatment or if they have a history of poor or uncertain adherence” [1–5]. LAI antipsychotics are associated with 47% fewer hospitalizations for schizophrenia and 44% fewer suicide attempts [6]. 

However, patient engagement with LAI antipsychotics is a long-standing problem. (We use the term ‘engagement’ rather than ‘adherence’ to acknowledge that, in contrast to oral medication adherence, LAI antipsychotic engagement requires meeting a professional at a time and place and accepting an intramuscular injection.) LAI antipsychotic engagement is more complex than oral medication adherence in that it involves attending an appointment with a professional, either at home or in a clinic setting, and consistently doing so at intervals often one, two, four, or 12 weeks apart. We define LAI engagement as attendance to appointments to receive an injection of an LAI antipsychotic at the prescribed frequency as per their treatment plan.

Patient LAI antipsychotic engagement is often inadequate for therapeutic effect, in part because patients often begin LAI therapy due to long-standing engagement problems [7]. LAI engagement rates commonly range between 60% and 85% which can result in patients with chronic schizophrenia spending a large part of the year with subtherapeutic drug levels [8, 9]. Non-adherence to LAI antipsychotics frequently precedes relapse [10]. 

Two randomized-controlled trials (RCTs) from European settings with government-led healthcare systems have shown that financial incentives improve patient LAI antipsychotic engagement, in keeping with other areas of medicine such as addiction psychiatry and cardiology [11–15]. Priebe et al. found incentivized patients engagement increased from 69 to 85% (vs. 67–69%, adjusted difference 11.5%, *p* = 0.003) over one year (*n* = 72 v 55) [12]. Similarly, Noordraven et al. found incentivized patients’ engagement increased from 76 to 94% (v 78–80%, adjusted difference 14.9%, *p* < 0·0001) over one year (*n* = 78 v 75) [13]. 

The attitudes of professionals, including psychiatrists, nurses, and other allied health professionals, towards financial incentives for LAI antipsychotics have been investigated previously, but these studies do not report whether professionals without personal experience of delivering financial incentives would support their implementation on the basis of the published empirical evidence. Focus groups conducted in 2008 (prior to both RCTs) revealed doubts about efficacy, concerns about funding, desire for clear policies, and ethical dilemmas, among psychologists, mental health nurses, consultant psychiatrists, patients and carers, and other stakeholders consulted [16]. These findings were based on participants’ assumptions about how patients would react to financial incentives but are outmoded now that there is evidence of how patients’ behavior changes when they are offered incentives. Subsequent research explored the experience of clinicians whose patients had been included in one of the RCTs. Reports from care coordinators, a community psychiatric nurses, and a mental health team managers revealed improved therapeutic relationships and reductions in patients’ symptoms, as well as instances where patients had increased their substance use, and a small number of cases where patients had sought larger amounts of money [17]. A survey found some divergent perspectives between patients and clinicians (including psychiatrists, psychologists, social workers and social psychiatric nurses) following the other RCT, but almost all agreed that their therapeutic relationships had improved [18]. 

Altogether using financial incentives appears to demand a balance of risks and benefits: there is clear evidence that financial incentives increase engagement, but policy makers in healthcare systems should also consider plausible evidence of some adverse outcomes of financial incentives such as occasionally asking for the LAI antipsychotic early in order to obtain the incentive sooner. The implementation of financial incentives into community mental health care is only likely if the balance is acceptable to frontline professionals. As such, any further progress towards using financial incentives in routine practice is only possible if professionals consider the existing evidence persuasive, or if they can identify practical means of mitigating adverse outcomes. This is particularly important given that those administering LAIs see themselves as advocates for patients, and collaborators in care when patients are compliant with their treatment plan [19]. 

Through semi-structured interviews with 14 professionals with responsibility for administering LAI antipsychotics, pre- and post a one-on-one informational session outlining existing empirical research based on a systematic review [20], this study aimed to evaluate how these empirical findings impacted professionals’ attitudes to financial incentives for LAI antipsychotics. A secondary aim was to investigate whether any additional concerns emerged in interviews which had been overlooked in our recent systematic review.

## Methods

### Recruitment

Participants were recruited from community mental health teams in an English National Health Service (NHS) mental health trust in the West Midlands in July and August 2023. The main criterion for inclusion was that the participant had some experience delivering LAI antipsychotic medication in a community secondary care context (i.e. a specialist mental health team providing care to patient who are not hospital inpatients), including early intervention and recovery teams, and being an allied health professional (not qualified doctors). Team leaders were initially contacted and further participants were recruited through snowball sampling until thematic saturation was achieved. Saturation was operationalized as when no new themes arose in 2 consecutive interviews [21]. 

Clinicians who only prescribed and did not administer LAIs were excluded from the study, as incentive policies are intended to increase the number of people accepting doses among those who have already consented and been prescribed the LAI. This approach to recruitment deliberately excluded prescribing psychiatrists. In this system psychiatrists do not administer LAIs, but prescribe in periodic face to face or remote appointments. By contrast, the participants we selected are more closely involved in the nuanced negotiation around the day-to-day administration of LAIs. Therefore, compared with psychiatrists, these participants are better able to describe the subtle interactions involved in patients accepting or declining LAIs and to raise concerns about the way financial incentives would interact with the actual practice of administering LAIs. Their role is most likely to be directly impacted by the provision of financial incentives; indeed in studies of financial incentives it has fallen to these professionals, rather than the psychiatrist, to administer the incentive. Limitations implied by this are discussed below but future research may consider exploring prescriber perspectives specifically.

### Interviews

A topic guide was developed based on a literature review (see appendix). Interviews were conducted over Microsoft Teams in July and August 2023 by MM and NH who are both psychiatrists. Initially, participants were invited to describe their experience with LAI antipsychotic treatment, their experience of engagement and their approach to maximizing engagement. After roughly 15 min interviews were paused and an informational presentation was given, outlining recent research on financial incentives. This approach is in keeping with Andrade’s guidance on assessing knowledge, attitudes and practice in psychiatry [22]. Then the interviews resumed and participants responses and reflections were elicited. During that period we were able to identify and correct any misunderstandings of the data. Participants were asked to rate their support for financial incentives on a scale from 1 to 10 where 10 is the most support before and after the informational presentation. Participants received a £25 voucher for taking part.

### Informational presentation

Participants were shown a brief presentation outlining key findings from a recent systematic review of financial incentives for LAI antipsychotic engagement (Table 1). Each slide included a question, relevant information, and a reference. The particular content of the slides related specifically to the key controversies arising in the systematic review [20]. Each presentation was delivered by authors of a systematic review who expanded on each point to add context and seek to address any misunderstandings. Specifically, the limitations of the past literature were highlighted. For example, participants were told that more LAIs were accepted with financial incentives than in the control group, but they were told that the sample size was too small to know whether this reduced relapse. Likewise, they knew there were two different RCTs with slightly different methods and different results but broadly similar positive results. Finally, participants were told that there was no rebound effect after withdrawing the incentive (*Will patients refuse* LAI antipsychotics *if their cash rewards stop?*) but it was clarified that there was no lasting positive effect either; we chose to emphasize the absence of a rebound effect because our concerns about rebound effects are more commonly reported in the literature than assumptions about habit formation [20]. Through this discursive presentation approach participants were able to gather a nuanced view of the literature.

**Table 1:** Research findings shared with professionals

Heading	Information	Ref
***(none)***	Some doctors have tried giving patients £20 in cash every time they get their LAI antipsychotic. The patients know in advance and get it every time they get their LAI antipsychotic. They are given it immediately after the injections is given. If they miss their appointment, they miss out on the £20.	[12, 13]
***Will it work?***	When patients who missed ¼ of their LAI antipsychotics were offered cash rewards, missed doses reduced by 80%.	[12, 13]
***Will patients see me as a cash machine?***	16% of professionals said relationships were worse but 30% of professionals reported better relationships.	[17, 18]
***Will patients refuse*** **LAI antipsychotics** ***if their cash rewards stop?***	After cash rewards were stopped patients were no more likely to miss their LAI antipsychotic than patients who never had incentives.	[23]
***Do patients feel insulted or manipulated?***	76% of patients tell researchers that they like having cash rewards because they feel like they are being celebrated for doing the right thing. 41% said they liked having more money to spend.	[18, 24]
***Is this a waste of NHS money?***	LAI antipsychotics are already very expensive, costing hundreds of pounds, some more than £200. If offering somebody £20 means they actually take it then it is small change compared to the total cost of treating somebody with an LAI antipsychotic.	[25]
***Will patients lie about their adverse effects?***	There’s no evidence of that as a switch to another LAI antipsychotic will not reduce incentives, but they might put up with minor discomfort better, saying “the money makes it better”.	[26]
***Will patients spend it on drugs?***	21/73 were reported to have used money to buy cigarettes or drugs. But others reported better engagement with drug and alcohol misuse services.	[17]

### Analysis

Interviews were audio recorded and transcribed verbatim by MM and NH. Both NH and MM read all transcripts and developed a coding frame. Both NH and MM coded all interviews. Themes and codes were clarified through an iterative process. In the secondary analysis NH and MM coded all studies against the themes raised in the systematic review. Any new codes which emerged in two or more interviews were added to the coding frame. The number of interviews mentioning each theme before the information presentation was counted. The same counting exercise was conducted for themes mentioned after the information presentation. If a theme was mentioned both before and after the information presentation by the same participant, then it was counted twice in total.

### Ethics and data availability

Ethics approval was obtained from the Biomedical Sciences Research Ethics Committee at University of Warwick. Data were anonymized prior to analysis. Transcripts are not openly available online due to sensitivity but are available from the authors upon reasonable request.

## Results

### Participants

Fourteen community mental health team professionals in the West Midlands region of England were recruited before saturation was achieved. Participants reported between 1 and 23 years of experience providing LAI antipsychotic care (mean = 9.5 years). 11 worked in Early Intervention and 3 worked in longer term ‘Recovery’ teams [27]. One current recovery nurse had also worked in Early Intervention previously. Twelve had a nursing background although one of these primarily worked as a practitioner in cognitive behavioral therapy for psychosis practitioner now. Two were occupational therapists.

### Thematic analysis

Table 2 outlines the results of the thematic analysis.

**Table 2:** Results of thematic analysis

Topic	Second Order Themes	First Order Themes
**Non-Engagement**	**Causes**	Forgetfulness
		Chronic psychosis symptoms
		Anxiety
		Adverse effects
	**Prevention**	Persuasion
		Prompting
		Formal policies
		Positive experiences
**Baseline Perceptions**	**Advantages of Financial Incentives**	Increased LAIA adherence
		Overcome forgetfulness
		Avoid relapse
		Efficient use of professionals’ time
		Positive patient experience
	**Concerns about financial incentives**	Attitudes to treatment
		Coercion
		Storing money
		Cessation of incentives
**Responses to Informational Presentation**	**Persuasive evidence**	Increased LAIA adherence
		Better overall engagement
		Small cost of LAIA
		Patients feel rewarded
	**Ongoing challenges**	Ensuring informed consent
		Public perceptions
		Practicalities
	**Misunderstandings**	Reduced relapse
		Skeptical of drug and alcohol findings

### Non-engagement with LAI in the community

#### What causes LAI non-engagement?

Participants reported that forgetfulness and symptoms of chronic psychosis combine with adverse effects and anxiety to lead to non-engagement. Non-attendance was primarily attributed to forgetfulness rather than strongly held preferences. Forgetfulness was linked to lifestyle factors such as a lack of routine: “some patients can be quite disorganized, lack time, normal structure and routine to their daily lives that they may miss appointments” (interview 2). Participants emphasized how patients gave mundane reasons for missing treatment: “it might be ‘I’m busy’. It might be ‘I have no funds to get there’. It might be that they’re away on that given day” (interview 11). One participant suggested patients may not attend appointments “if they’re not morning people” (interview 14).

Symptoms of chronic psychosis such as lack of insight - “patients don’t believe they have a mental illness so they don’t see the need” (interview 7) – or auditory hallucinations may make attendance difficult: “Yesterday he just slept in because the voices were bad and it was a bit too much for him.” (interview 6).

Patients may miss injections because “Sometimes they’re scared of it” (interview 8) or because of concerns about adverse effects (interview 3) but often give a different “excuse” (interview 9), which makes the exact interplay of forgetfulness, symptomatology, anxiety and adverse effects difficult to establish.

#### How do professionals currently promote LAI engagement?

Existing approaches to overcoming non-adherence included persuasion, prompting, formal policies, and linking the injection routine with positive experiences. Participants emphasized that these different features all worked together.

Persuading patients to accept LAI antipsychotics was described as “psychoeducation around the benefits of the medication” (interview 1), “providing information about the benefits” (interview 4) and “thinking about how they presented before and how having this medication is going to prevent that” (interview 13).

Practitioners also “prompt” them (interview 8) and help them remember appointments:

We usually find people who are just not that organized writing it on their calendar. Or putting it in their phones. We tend to do text message or phone call reminders. Maybe the day before even, with some people. (interview 3)

Help with travel to the clinic included offering free bus passes or asking supportive relatives to drive patients. LAIs are often delivered in the patient’s home because it is “considerate” (interview 14). However, this makes it more difficult to ensure equipment sterility and privacy and is resource-intensive for the service: “going to their homes… it’s time consuming, it’s draining for yourself and I suppose it’s staff resources as well of having to going out to the person… lots of energy, lots of time.” (interview 4).

‘Road to Recovery’ groups were cited as helping patients gain confidence about leaving the house and participants similarly reported providing informal “graded exposure” to build confidence (interview 14) [28]. Regularly monitoring side-effects also ensured that professionals are alert to the need to switch to a different LAI: “We do try and change medications, especially when people are coming in and we can see that they’re gaining weight, that they’re perhaps kind of feeling more tired. So we do use the LUNSERS to monitor side effects.” (interview 3).

Other participants talked about the ways they helped patients look forward to the day they received their LAI by linking it with positive experiences. Some patients make their LAI into a day out - “quite a lot that will go into town after” (interview 6) – and other professionals members talked about “bargaining” (interview 9) and “bribing” patients to persuade them to accept their LAI:

You’d call it, like, a bribe because we could collect, like, petty cash expenses … We can go get something to eat. We’ll buy you a sandwich. We’ll buy you a cake and a coffee. Something like that. … you sort of go in with whatever you can to sort of engage that person to spend a bit more time with you, so at least you can chat to them and have that time to maybe convince them to have the depot. (interview 10)

### Baseline attitudes towards financial incentives

#### How did professionals view financial incentives for LAI engagement?

At baseline, support for financial incentives was rated out of 10. The median reported score was 5 out of 10 with participants commenting “I would need to know more about the research” and “I could see advantages and disadvantages of it”. Baseline support ranged from 3 out of 10 to 10 out of 10. All professionals discussed positives and negatives of a financial incentive program, weighing and comparing them.

#### What advantages of financial incentives were identified at baseline?

Financial incentives were predicted to increase engagement with medication, improving patients’ mental health, and save the service money. A professional who rated their baseline support at 3 out of 10 nevertheless acknowledged “I’ve got a couple of people on my caseload who refuse medication, become unwell, and probably would be open to that idea and it would actually keep them well” (interview 12). Financial incentives could however overcome disorganization-driven engagement “for the people who are just a bit forgetful … It might make people be a bit more regular, prioritize it, you know”. (interview 8).

The priority for the goals of treatment was to avoid relapse which clinicians saw as important for the patient and the health service: “that’s going to be a massive cost to their own health and to society”. (interview 6) The immediate costs of missed appointments were seen as problematic: “if they don’t attend, it’s a massive waste of staff time” (interview 1). The direct benefits of cash were noted as “something to look forward to” (interview 13) and *“extra money to help with the kids.”* (interview 2).

#### What concerns about financial incentives were raised at baseline?

Concerns related to patient attitudes to treatment, ethics, and practicalities.

There were worries that financial incentives would not “build the therapeutic relationship” (interview 12). Worse, participants could be “angered if they genuinely can’t make the appointment and then they feel like they’ve lost their £20” (interview 8). Others argued “straight away it’d go on drugs” (interview 10) and suggested that the extra money would lead to worsening mental health “There will be a percentage where the mental health deteriorates because they’re then going to go and use more substances”. (interview 11)

Ethical concerns were raised about “coercive” power dynamics (interview 12) or “bribery” (interview 3). The nuances of consent were discussed:

I feel like it should still be a choice and it is a choice but then you’ve got those vulnerable patients who would jump at that chance (to receive money). (interview 13)

There were fears that the public might consider incentives wasteful, asking “where does the funding come from? … is it coming out of the taxpayers’ money?” (interview 4).

Practical questions relating to implementation included, “how do you keep the money safe?” (interview 6), wondering how long the incentive would last for and whether it would be consistent across different services where LAIs are given, including primary care services (interview 3).

Others were concerned about perverse effects if patients disengage in order to become eligible for an incentive (interview 7) or disengage following incentive cessation (interview 14). One participant raised a contrasting concern: “no one’s ever going to want to come off the depot” (interview 2).

### Perspectives after the research presentation

#### Did overall support change?

After a presentation describing the existing research, participants had chance to discuss the research and were asked a second time to rate their own support of out of 10. The new median support was 6.75 out of 10 (range 4–10). Mean support increased by 1.1 out of 10 (range 0–3). One participant who was largely skeptical about incentives reported a score of 4 but emphasized openness to the approach: “if I was informed it was something we were gonna start doing I wouldn’t be entirely against it and questioning my job” (interview 12).

#### Which evidence did participants’ find persuasive?

The most persuasive data suggested incentives led to increased LAI engagement during the intervention, stronger therapeutic relationships and low levels of substance abuse. Compliance was linked with improved outcomes: “because they were on the depot they were feeling a bit better, and maybe their recovery then improved, and then they were more well, and then able to engage better” (interview 14). Another put it this way:

Using that opportunity to get the depot to continue to build on that relationship and to encourage further engagement and other therapies or interventions that we do, it’s a, it’s a foot in the door. (interview 2)

The size of the cost of the incentive was considered “small change” (interview 4) and “tiny” (interview 6) compared to the cost of LAI treatment. One commented, “It’s a bargain!” (interview 8).

Incentives were conceptualized as rewards and that patients were “being rewarded for taking it, ‘celebrated’ even, that was the word, wasn’t it? And that that was probably a nice way of kind of seeing it” (interview 4).

When I was a child I had my vaccinations. And then after you would get a lolly and a sticker and you know, different things because then it wasn’t as bad. (interview 7)

#### What ongoing challenges did professionals anticipate?

Ethical and political issues were discussed, but practical challenges were considered in greater detail, several offering potential solutions.

After discharge from mental health services, many patients continue LAIA treatment under primary care teams, leading several participants to wonder whether engagement would decrease if primary care was unable to offer incentives (interview 1). Discharging patients to primary care is a point where patients were seen to disengage from LAI treatment and suggested that if incentives for LAIs continued in primary care then the change might be better tolerated by patients (interview 3).

There was added concern about how to handle cash “safely” (interview 6) and suggesting that a “prepaid voucher” would be more appropriate (interview 2). While some feared that incentives would not be effective, others were concerned that it would be harder to persuade patients to stop LAI treatment, despite acknowledging that services have not previously had difficulty persuading patients to cease LAI treatment (interview 3).

Two participants spontaneously commented that they thought more research was needed. When asked about what would persuade them and their colleagues, participants commented on cost-effectiveness – “how much it would actually improve like the NHS, improve services, cut costs?” (interview 13) – and ensuring research was applicable to their own local population.

Ethical concerns were reiterated more strongly after the presentation. One participant objected that incentives threaten informed consent: “We need to gain informed consent from an individual when we give this medication” (interview 11).

Equity and fairness of incentives were discussed, as it was seen as being unfair on those with good engagement if only those who engage poorly received incentives (interview 13). Another said that “politics” would be a problem (interview 6): “they’re getting more money, they’re already on benefits, all that kind of rhetoric that goes on and on and on really.” (interview 6).

#### What results did professionals misunderstand?

After the informational presentation covered eight new topics, some participants had misunderstood one of them and we immediately corrected these misunderstandings. Some appeared to have initially misunderstood the finding related to effect persistence after removal of the incentive: “even though the cash incentives stop, people are more likely to continue with their depot” (interview 1) when in fact, following cessation of incentives, engagement returned to baseline and was no different from the control group.

Participants emphasized the finding that LAIA engagement increased but also extrapolated that this would reduce relapses: “We’ll have more people on more antipsychotics, and would have less relapses.” (interview 6) The putative reduction in relapses was linked to wider health system finances: “if it negates one one-month hospital admission, then it’s probably become extremely cost effective” (interview 12).

There was skepticism about the research finding that few study participants spent the money on drugs and alcohol, especially raising the methodological concern that drugs and alcohol use is a “hard one to measure” (interview 11). Another raised concerns that sample sizes in existing studies had been too small to show an increase in LAIA adherence, although in fact a statistically significant result had been found (interview 1).

### Comparison with systematic review

**Fig. 1:**
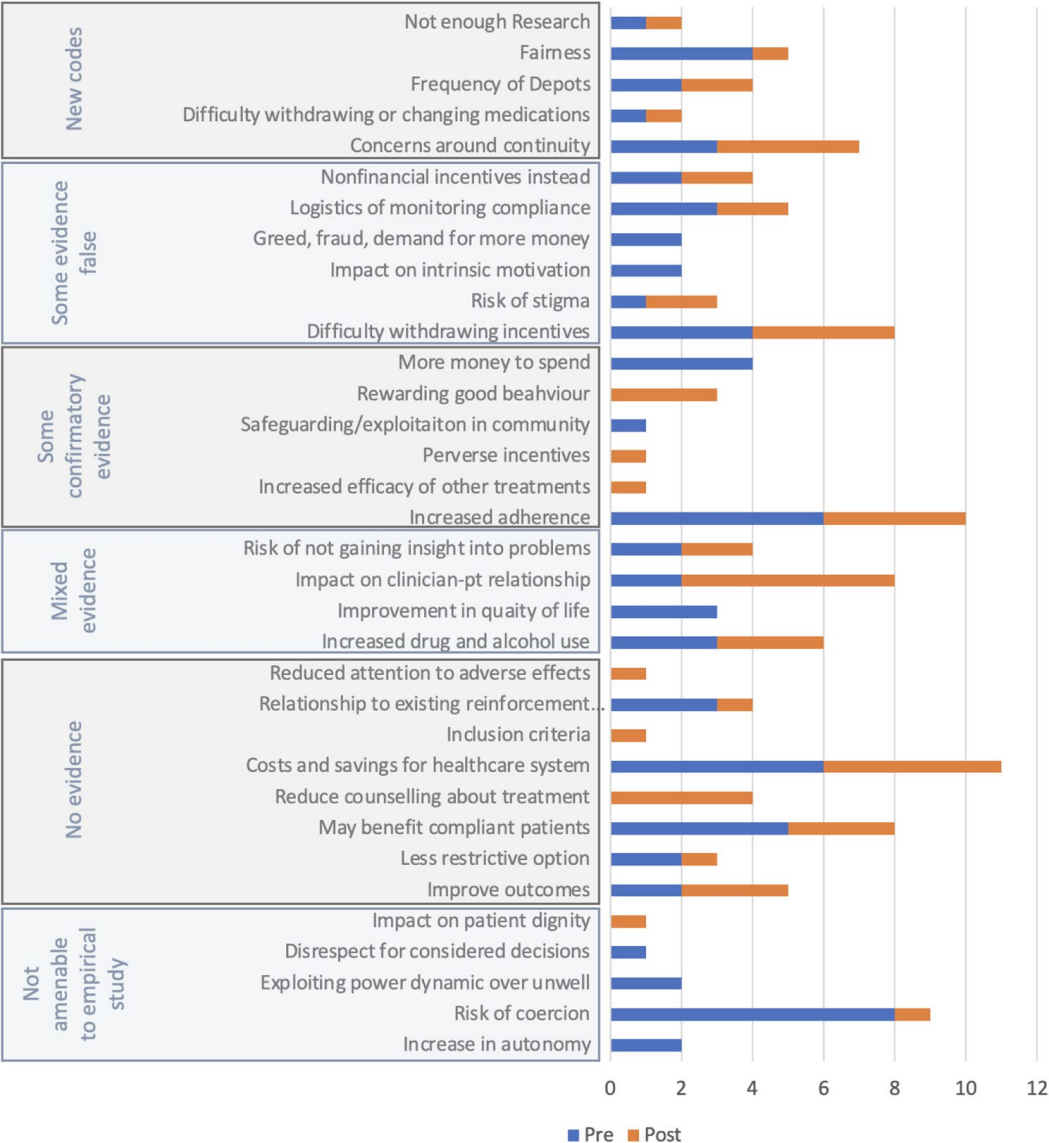
How many participants mentioned each theme from the systematic review before and after information presentations?

Professionals raised 29 of the 40 topics in a recent systematic review [20]. Fig. 1 shows which topics were touched upon before and after the information presentation.

Five new themes were identified which had not been identified in the systematic review (Concerns around continuity; Difficulty withdrawing or changing medication; Frequency of LAIAs; Fairness; and, Not enough research). 11 themes from the systematic review were not mentioned in any interviews: flexibility, appropriate incentive size, personalization, increased demand for psychopharmacology, penalizes adherence, sense of entitlement, habit formation, financial dependence, may not work, may not help forgetful people, supplants social support.

Although the idea of unfairness emerged, none specifically suggested that good adherence was penalized. Participants also suggested patients taking LAIs would not wish to stop taking their LAI, but no participants said overall demand for antipsychotics would increase.

Figure 1 shows that in the pre-information interviews, participants brought up topics which were rated ‘not amenable to empirical study’ in the systematic review, such as the idea incentives increase autonomy or may be coercive. After the information session they prioritized practical issues like continuity and the withdrawal of incentives, the importance of increasing adherence, therapeutic relationships, and health economics.

## Discussion

The impact of empirical research on professionals’ attitudes to financial incentives for LAI antipsychotics was evaluated through interviews with 14 professionals with responsibility for administering LAI antipsychotics, conducted before and after informational sessions outlining the existing literature on systematic reviews. Participants’ support for financial incentives increased from median of 5 out of 10 (range 3–10) to 6.75 (range 4–10) after exposure to the evidence.

Patient non-engagement with LAI antipsychotics was attributed to forgetfulness and symptoms of chronic psychosis combined with adverse effects and anxiety. Existing approaches to overcoming non-adherence included using persuasion, prompting, formal policies, and linking LAIs with positive experiences. Before hearing about recent research, participants anticipated that incentives would increase engagement with medication, improving patients’ mental health, saving the service money, and benefiting patients’ finances. Concerns related to patient attitudes to treatment, ethics, and practicalities. With respect to existing research, the most persuasive evidence was that incentives led to increased LAIA engagement during the intervention, leading to stronger therapeutic relationships and low levels of substance abuse. However, professionals remained alert to ethical and political questions, and considered practical challenges carefully, proffering practical solutions.

### Comparison with the literature

This interview study has largely validated the findings of a previous systematic review [20]. 29/40 codes in the systematic review were brought up during interviews (see Fig. 1). Five new codes were added, which mostly related to the practical implementation of financial incentives, indicating that participants in the present study considered practical issues more carefully than participants in previous studies. This finding is perhaps unsurprising because in previous studies the efficacy of financial incentives was the main topic of consideration, whereas in the present study financial incentives were shown to be an effective approach requiring further consideration of implementation issues.

Although previous studies have explored the attitudes of professionals (including psychiatrists, psychologists, and mental health nurses) before trials were conducted, and professionals who have been part of trials, this is the first evaluation of the attitudes of professionals who have been told financial incentives for LAI antipsychotics are a proven effective means of increasing engagement [16–18]. This evidence of efficacy was important for participants who inferred that it would prevent relapses, avert hospitalizations, and reduce total healthcare costs. When asked what would strengthen the case for financial incentives, several professionals called for larger studies powered to demonstrate reduced hospitalizations. Previous health economic evaluations have been unable to draw these conclusions so larger studies are merited [29]. 

A recent systematic review of acceptability of financial incentives across healthcare settings identified only 9 studies, including two previous studies in the context of long-acting injectable antipsychotics, and have had mixed results [30]. For example, 12 professionals shared their views on an incentive program for anti-retroviral therapy adherence in the USA, reporting a positive overall evaluation but acknowledging difficulty withdrawing the incentives at the end of the study [31]. In contrast, 18 professionals shared their views on offering incentives for childhood immunizations in the UK overall broadly opposing the approach as commodification [32]. The present study shows that providing an update on the scientific evidence can help professionals refine their thoughts on financial incentives and other studies may find a similar approach beneficial.

### Strengths and limitations

Compared with treatment as usual, financial incentives have proven potential to increase LAI antipsychotic engagement but their potential for practical implementation has not been fully explored until now [12, 13]. The use of interviews in this study allowed participants to express their nuanced reflections and think aloud. Participants were frontline professionals with extensive experience of the interpersonal and systemic dynamics around financial incentives, and therefore were best placed to comment on practical and relational aspects of the proposed intervention. Interview studies do not formally allow participants to confer about their perspectives, but in a real-world implementation of financial incentives ethical issues are likely to be discussed in the coffee room.

This study relied on a relatively small number of participants, although saturation was reached. Participants were all from the same region of England and views internationally may differ, although in the context of British mental healthcare there is nothing particularly unusual about the mental health teams the participants were drawn from; international replication would be valuable. Likewise, no psychiatrists were included in the sample because they prescribe but do not administer LAIs in the study settings. To comment on our reflexivity, three of the authors are psychiatrists who have prescribed LAIs but our interactions with colleagues have engendered in us epistemic humility regarding the complex and nuanced relational work carried out by our colleagues who administer the medications and consider the prescribers’ perspective less complex than that of the person who administers it. However, it is plausible that other psychiatrists would provide additional insights and future scholarship may explore whether psychiatrists have wider ideas or concerns regarding the impact on patients of financial incentives.

Our snowball sample included people who were particularly interested in discussing the topic with us, or that participants referred us on to people who shared characteristics with them, but the presence of diverse perspectives suggests this recruiting strategy did not result in a biased population. It may be that people who prefer not to discuss ethical issues were underrepresented and as such numeric estimates in this study should be interpreted with care.

Similarly, participants may have assumed that the investigators had an agenda regarding financial incentives. Perhaps it is most likely they would have assumed investigators supported the introduction of financial incentives because financial incentives are not currently available, so only advocates would investigate their potential. However, several participants were happy to voice opposition or concerns, suggesting social desirability bias was limited. However, recency bias may mean that the presentation had an outsized effect on the second measurement of acceptability which was taken during the same session as the informational presentation and therefore the positive effect may diminish over time.

### Implications for policy and practice

We found professionals suggested several ways financial incentive regimes could be implemented more smoothly than in previous research. Whereas RCTs only studied participants with low baseline engagement, professionals suggested that this would be unfair and could perversely incentivize non-engagement; instead financial incentive regimes should offer incentives to all patients receiving LAI antipsychotic therapy [12, 13]. Similarly, no previous study has considered continuity between secondary care and primary care and we suggest financial incentives should be given regardless of clinical context, although perhaps smaller incentives could be given in primary care as patients are likely stabilized on medication after discharge from secondary care. Gains in fairness and engagement, not to mention programmatic simplicity, would likely outweigh the cost of additional incentives, but an empirical study and economic analysis is required.

Concerns about managing cash were also raised. We estimated a sum of GBP20 by assuming the incentive would remain in the same ratio to the National Minimum Wage seen in the RCTs [20]. We did not ask specifically about whether this was the right amount of money, but no participants suggested it was excessive or insufficient. However, participants were concerned about how they would store the cash required. This fear was borne out in the RCTs where professionals had become anxious when they forgot to bring cash on home visits [17]. Incentives in the form of a pre-payment card or voucher were proposed as alternatives to cash, with the added benefit of being less easily exchanged for drugs. In a systematic review of incentives for survey completion, cash was more effective than vouchers (RR = 1.19) [33]. However a head-to-head study comparing vouchers and cash in the context of contingency management of cocaine addiction found no difference in cocaine use among 144 participants [34]. Future scholarship should explore whether vouchers and cash have the same impact on behavior among people experiencing psychosis.

Either way, an incentive design which reduces the need to manage petty cash would be preferred by professionals. In other contexts, lotteries have been used to provide financial incentives, allowing awards to be delivered at a later date, but the larger magnitude of lottery prizes may increase the risk of substance use to excess [11]. One recent proof-of-concept study showed that incentives for oral antipsychotic adherence could be delivered via an app, suggesting digital distribution of incentives could be promising [35]. 

Financial incentives may always be ethically contentious, as illustrated by the most strongly opposed participants. Several ethicists have discussed whether it is wrong to offer incentives to patients for adhering to antipsychotic medication. In this study some participants said that professionals already offer incentives informally, such as going out for lunch or having coffee. Participants also responded well to the concept of incentives being ‘rewards’ or ‘celebrations’. Following Noordraven et al.’s RCT, 76% of patients who had received incentives endorsed the concept “It is good to reward good behavior with money” whereas only 38% of professionals endorsed that notion [18]. If professionals and the public accept that incentives are ‘rewards’ then it may be easier to placate public opinion, which participants also identified as a significant challenge.

Table 3 summarizes how adjustments to the financial incentive policy can address concerns, and outlines the required future research.

**Table 3:** Alternative policies to address professionals’ concerns

Concern	Alternative Policy	Evidence Needed
Attitudes to treatment	Present incentive as a prize or reward, not payment	Does ‘reward framing’ avoid transactional relationship?
Therapeutic relationship	Other professionals, not care coordinator, to give incentive	Is separating roles practically feasible?
Coercion	Ensure informed consent taken prior to providing the incentive	Can doctors consent patients without discussing incentives?
Storing money	Use vouchers	Is the effect of vouchers and cash different?
Cessation of incentives	Continue but taper incentives over years	Does tapering incentive after 2 years decrease LAIs?
Unfair on good adherers	Provide incentive to all, not just poor adherers	Full health economic evaluation required
Public perceptions	Emphasize reduced hospitalization	Full health economic evaluation required
Concerns about drug and alcohol use	Use vouchers	Are vouchers are sold and does drug use increase?
Discharge to primary care	Continue incentive after discharge and taper	Will primary care professionals support use of incentives?

## Conclusions

Many of our participants were enthusiastic about the prospect of using financial incentives to reduce relapse suggests this line of research remains promising. The concerns raised by other participants highlight the need for further research including evaluation of whether different structures of incentive regimes can address concerns. In this paper we have proposed that future studies investigate financial incentive regimes which include all patients on LAI antipsychotics, not just disengaged patients or secondary care patients, although we acknowledge the magnitude may vary between primary care and secondary care contexts. The behavioral impact of alternatives to cash, such as app-based payments, vouchers, and pre-payment cards should be investigated further. Patients’ preferences with respect to the delivery of incentives should be explored. Future RCTs should aim to be powered sufficiently to identify whether the expected reduction in hospital admissions is achieved, as this was seen as the most important measure.

## Supplementary Information

Supplementary Material 1.

## Data Availability

All interview transcripts are available on request. Uploading these data publicly is not appropriate as transcripts include details of conversations with patients.

## Abbreviations

LAI: Long-Acting Injectable
RCT: Randomized-Controlled Trial
NHS: National Health Service
USA: United States of America
GBP: Great British Pound
RR: Risk Ratio
