# Supplementary material for: Financial incentives and long-acting injectable antipsychotics engagement: community mental health professionals’ perspectives
Source: BMC Psychiatry. 2025 Aug 18;25:791. doi: 10.1186/s12888-025-07165-9 (PMC12359960; doi:10.1186/s12888-025-07165-9)
Supplement: Supplementary file 1 — Supplementary Material 1. [file 12888_2025_7165_MOESM1_ESM.docx]

**Appendix 1: Topic Guide**

**Study Title:** Mental Health Staff Attitudes to Incentives for Antipsychotic Depots

**Investigator(s):** Dr Nathan Hodson, Dr Madiha Majid, Professor Ivo Vlaev, Professor Swaran Singh

**Opening:** introduction and consent forms. (word document)

**Part 1:**

What is your role?

How long have you been involved in giving antipsychotic depots?

Talk me through the process when you give antipsychotic depots.

Is there anything difficult about this process?

Do patients attend their appointments to receive the antipsychotic depots? If yes or no, why do you think that is?

Is there anything you or the service does to encourage patients to attend depot appointments?

Some people have tried giving patients £20 in cash every time they get their depot. The patients know in advance and get it every time they get their depot. They are given it immediately after the injections is given. If they miss their appointment they miss out on the £20. What do you think about that?

In favour to against 0 to 10?

What do you think would be the advantages of that?

What do you think would be the potential problems with it?

**Part 2:**

Here are the key facts about giving patients cash rewards for taking antipsychotic depots:

1. Will it work? When patients who missed ¼ of their depots were offered cash rewards, missed doses reduced by 80%.
2. Will patients see me as a cash machine? Many staff reported improved relationships with patients and patients increased participation with other areas of therapy.
3. Will patients refuse depots if their cash rewards stop? Evidence shows that patients whose cash rewards were stopped were no more likely to miss their depot than patients who never had incentives.
4. Do patients feel insulted or manipulated? No, 76% of patients tell researchers that they like having cash rewards because they feel like they are being celebrated for doing the right thing. 41% said they liked having more money to spend.
5. Is this a waste of NHS money? Depots are already very expensive, costing hundreds of pounds, some more than £1000. If offering somebody £20 means they actually take it then it is small change compared to the total cost of treating somebody with an antipsychotic depot.
6. Will patients lie about their adverse effects? There’s no evidence of that as a switch to another depot will not reduce incentives, but they might put up with minor discomfort better, saying “the money makes it better”.
7. Will patients spend it on drugs? Patients who want drugs can buy drugs already so there is no evidence that small incentives increase drug use. In fact, some patients may stay well enough to avoid drugs and may not need to self-medicate.

**Part 3:**

Now that you have heard some key facts from the research, have any of your perspectives changed?

What did you think was the most persuasive fact you heard?

Were there any facts you didn’t believe or were sceptical of?

Do you feel reassured based on this data? In favour to against 1 to 10?

Is there anything else you would want to know about financial incentives?

What would it take to convince you to support cash rewards for depots? **OR** What do you think we would have to say to persuade colleagues to advocate for cash rewards for depots?
